# Estimation of 10 - year probability bone fracture in a selected sample of Palestinian people using fracture risk assessment tool

**DOI:** 10.1186/1471-2474-14-284

**Published:** 2013-10-05

**Authors:** Mai B Aker, Adham S Abu Taha, Sa’ed H Zyoud, Ansam F Sawalha, Samah W Al-Jabi, Waleed M Sweileh

**Affiliations:** 1 College of Graduate Studies, Public Health Program, An-Najah National University, Nablus, Palestine; 2 Division of Pharmacology/ Toxicology, College of Medicine and Health Sciences, An-Najah National University, Nablus, Palestine; 3 Division of Clinical Pharmacy, College of Medicine and Health Sciences, An-Najah National University, Nablus, Palestine

**Keywords:** FRAX, Osteoporosis, Palestine

## Abstract

**Background:**

The Fracture Risk Assessment (FRAX) tool has been developed by the World Health Organization (WHO) to calculate 10-year probability hip fracture (HP) or major osteoporotic fracture (MOF). The objective of this study was to assess the 10-year probability of MOF and HF among a selected sample of Palestinian people.

**Methods:**

A sample of 100 subjects was studied. Dual energy X-ray absorpitometry was performed to measure bone mineral density (BMD) which was then inserted into FRAX Palestine online WHO tool to calculate the 10-year probability of MOF and HF.

**Results:**

The median age of participants was 61.5 years and the majority (79%) were females. The median (interquartile range) of femoral hip BMD was 0.82 (0.76-0.92) g/cm^2^. The mean vertebral and hip T scores were -1.41 ± 0.13 SDs and -0.91 ± 0.10 SDs respectively. About one fifth of the sample (21%) had vertebral osteoporosis and 5% had hip osteoporosis. The median (interquartile range) 10-year probability of MOF and HF based on BMD were 3.7 (2.43-6.18)%, and 0.30 (0.10-0.68)% respectively.

**Conclusion:**

Osteoporosis is common among Palestinian people above 50 years old. Bone fracture prevention strategies and research should be a priority in Palestine. Using FRAX might be a helpful screening tool in primary healthcare centres in Palestine.

## Background

Osteoporosis is a progressive silent disease affecting bone mass and structure, leading to increased susceptibility to fractures [1,2]. The most common sites for osteoporotic fractures are the spine, hip, and wrist. Hip fractures (HP) are particularly devastating [3-5]. The World Health Organization (WHO) recently designed a tool to estimate the 10-year probability of HF and major osteoporotic fracture (MOF). This tool is called Fracture Risk Assessment Tool (FRAX) [6]. The tool, FRAX, is based on 11 risk factors plus the hip bone mineral density (BMD) if available. Each of the 11 factors used in FRAX tool provides some degree of independent information about fracture risk. These factors include age, sex, weight, height, a prior fragility fracture, parental history of HF, current tobacco smoking, long-term use of glucocorticoids, rheumatoid arthritis, other causes of secondary osteoporosis and daily alcohol consumption. Since osteoporosis is a multi-factorial disease, combination of risk factors with BMD produces the most effective risk assessment for MOF as opposed to assessment of any risk factor alone [7].

FRAX is intended for use in postmenopausal women and men over the age of 40 who have not taken osteoporosis medications. The tool is available online at (http://www.shef.ac.uk/FRAX/tool.aspx?country=52). Normal BMD or bone mineral content (BMC) is defined by the WHO as BMD or BMC score between ± 1 standard deviations (SDs) from the young adult mean, as measured by central (hip or spine) dual energy x-ray absorpitometry (DEXA) scan [8]. Osteopenia is clinically defined as a BMD score between -1 and -2.5 SDs and osteoporosis as a BMD score 2.5 SDs or more below the young adult mean. The National Osteoporosis Foundation (NOF) recently developed guidelines based on FRAX and currently recommends starting treatment in individuals with any one of the following criteria: (1) history of hip or vertebral fracture, (2) T-score ≤ -2.5 at femoral neck or spine, (3) T-score between -1.0 and -2.5 and 10-year probability ≥ 3% for hip fractures and ≥ 20% for MOF [9].

Palestine, or called the occupied Palestinian territories is located in the Middle East area. According to the latest report published by the Palestinian ministry of health (http://www.moh.ps/attach/441.pdf), Palestine has a total population of (4,168,858); (50.8%) are males and (49.2%) are females. Population pyramid shows that (40.8%) of the Palestinian population is under 15 years old, 14.7% is in the age group (0–4) years, and 2.9% is above 65 years. The natural increase of population was 2.9%, and the crude birth rate was (29.1/1,000) while fertility rate was 4.3. The five main health providers of health services in Palestine are Ministry of health (MOH), the United Nations Relief and Works Agency for Palestine Refugees in the Near East (UNRWA), Palestinian Non-Governmental Organizations (NGOs), Palestinian Military Medical Services (PMMS) and Private for profit organizations. The Palestinian MOH bears the heaviest burden, as it has the major responsibility. The NGOs sector operates more than 200 primary health care centres and general clinics in Gaza Strip and West Bank [10].

To the best of authors’ knowledge, no studies have been carried out in Palestine or in other neighbouring Arab countries using FRAX® to estimate the 10-year probability bone fracture. Therefore, this study was carried out to measure the BMD and calculate the 10-year probability hip and MOF in a selected sample of Palestinian men and women older than 50 years.

## Methods

### Study design and setting

This study was a descriptive analytical study carried out at Al-Rahmah center which is a nongovernmental charitable organization that provides medical services for the general public. It includes outpatient specialist clinics, pharmacy, radiology and laboratory departments. It has a relatively high workload and it is the only center that provides DEXA in Nablus district, Palestine.

### Sampling method and ethical consideration

Data collection process took place during April – June, 2012. One of the investigators visited Al-Rahmah center daily and stayed there from 9 am to 1 pm to recruit participants. Potential candidates were approached and were invited to participate. Males or females above 50 years who did not have osteoporosis and were not using osteoporotic medication, except for calcium and vitamin D, were included in the study. Those having recent osteoporotic fractures were excluded.

Ethical approval was obtained from Institutional Review Board (IRB) at An-Najah National University. In addition, approval from Al-Rahmah clinic administration, and consent forms from participants were also obtained. Participants were assured privacy and confidentiality of data.

### Data collection tool

The questionnaire used consisted of 1) socio-demographic information including sex, age, educational level, and marital status, 2) anthropometric measures including height, weight, and BMD, 3) medication and medical history of the participants and 4) dichotomous risk factors of FRAX tool.

Bone mass density was obtained by performing DEXA using Hologic DEXA machine at Al-Rahmah clinic radiology department. The hologic DEXA machine was set for white Mediterranean as reference value. Further classification was made based on the WHO classification for osteoporosis (BMD value is - 2.5 SD or more below the mean for young adult mean) and osteopenia (BMD value between -2.5 SD and -1 SD) to classify the subjects according to vertebral and hip osteoporosis. Weight and height were also recorded. Height was measured also by tape measure. When using FRAX/ Palestine tool, the DEXA result was entered as BMD measurement. The online FRAX tool calculates the 10-year probability of both HF and MOF.

### Data analysis

The independent variables included in this study were: age (above 50 years); sex; marital status (single, married, widowed, and divorced); parity and use of oral contraceptives (OCT) in females; weight (kg) and height (meter) to calculate body mass index (BMI); level of education (illiterate, basic education (1^st^ to 10^th^ grade), high school education, and college); administered medications, and chronic illnesses. Other investigated variables such as history of previous fracture, history of parental fracture, alcohol consumption, tobacco smoking, history of glucocorticoids use, rheumatoid arthritis, other secondary causes of osteoporosis, and exercise were presented as dichotomous variable. Secondary osteoporosis is present if the patient has a disorder strongly associated with osteoporosis. These include type I (insulin dependent) diabetes, osteogenesis imperfecta in adults, untreated long-standing hyperthyroidism, hypogonadism or premature menopause (<45 years), chronic malnutrition, mal-absorption or chronic liver disease. The outcome variable in this study was the 10-year probability HF and MOF calculated by the WHO FRAX tool for Palestine.

Statistical analysis was conducted using Statistical Package for Social Sciences (SPSS) version 16.0 for Windows. Normality was tested using Kolmgorov-Smirnov test. Descriptive analysis for continuous variables was performed with mean and SDs or median and (interquartile range). Frequencies and percentages were calculated for categorical variables. Correlation was used to test relationship between BMD and 10-year probability of HF or MOF. Mann–Whitney U test was used to test association between groups for variables that were not normally distributed. Differences were considered significant if the P-value was less than 0.05.

## Results

### Demographic and clinical characteristics of the participants

**Figure 1:**
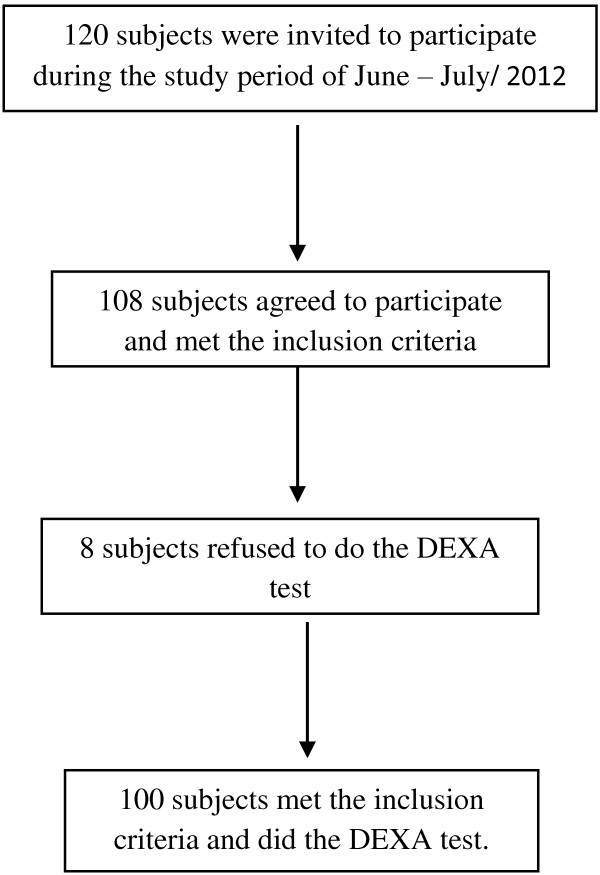
Number and response of subjects who were invited to participate.

One hundred and twenty people were invited to participate in the study, 12 refused to participate while 8 did the interview but refused to do the DEXA test, giving a net total sample of 100 subjects (Figure 1). Demographic and clinical characteristics of the participants are shown in Table 1. Age showed positive skewness with a median age (interquartile range) of 61.5 (55–67) years. The majority (79%) of participants were females. The mean ± SD of BMI of the study sample was 32.20 ± 4.69; for males was 28.92 ± 4.91, and for females was 33.07 ± 4.26. Nineteen percent of the participants were current tobacco users and 38% exercised routinely at least 30 minutes a day three times a week. More than half of female participants (53.16%) reported using oral contraceptives in the past. Twenty two percent of the participants had used oral corticosteroids. Twenty-two percent suffered from previous fracture and 15% had at least one parent with previous HF.

**Table 1:** Demographic and clinical characteristics of the participants

**Variable name**	**Statistics Mean ± SD or Median (interquartile range) or N (%)**
**Age (years)**	61.5 (55–67 )
50 < Age ≤ 65	72 (72%)
65 < Age ≤ 90	28 (28%)
**Gender**	
Male	21 (21%)
Female	79 (79%)
**Marital status**	
Married	91 (91%)
Others (single, widowed, or divorced)	9 (9%)
**Parity (for females)**	
Nulliparity	8 (10.13%)
Have ≤ 6 children	29 (37.05%)
Have > 6 children	42 (53.16%)
**Education**	
Illiterate	18 (18%)
School educated	58 (58%)
Achieved university degree	24 (24%)
**Current tobacco use**	
Yes	19 (19%)
No	81 (81%)
**Body mass index**	32.20 ± 4.69 g\cm^2^
For males	28.92 ± 4.91 g\cm^2^
For females	33.07 ± 4.26 g\cm^2^
**Exercise**	
Yes	38 (38%)
No	62 (62%)
**Previous fracture**	
Yes	22 (22%)
No	78 (78%)
**Previous parents’ hip fracture**	
Yes	15 (15%)
No	85 (85%)
**History of OCT use among females**	
Yes	37 (53.16%)
No	42 (46.84%)
**History of oral corticosteroid use**	
Yes	22 (22%)
No	78 (78%)
**Secondary osteoporosis**	
Yes	15 (15%)
No	85 (85%)
**Diabetes mellitus**	
Yes	29 (29%)
No	71 (71%)
**Cardiovascular diseases**	
Yes	60 (60%)
No	40 (40%)
**Gastrointestinal tract disorders**	
Yes	32 (32%)
No	68 (68%)
**Rheumatoid arthritis**	
Yes	25 (25%)
No	75 (75%)

*Abbreviations*: *SD* standard difference, *OCT* oral contraceptives.

### Dual energy X-ray

The results of BMD had a median (interquartile range) of 0.82 (0.76 - 0.92). The mean vertebral T score was -1.41 ± 0.13 SDs and the mean hip T score was - 0.91 ± 0.095 SDs. Based on the WHO criteria, 21% of the participants had vertebral osteoporosis, 29% had vertebral osteopenia and 50% were normal. Based on hip T scores, 5% had hip osteoporosis, 23% had hip osteopenia, and 72% were normal. Taken altogether, 23% had osteoporosis whether hip or vertebral (Table 2).

**Table 2:** **Results obtained from Dual Energy X**-**rays** (**DEXA**)

**Variable name**	**Statistics Mean ± SD or Median (interquartile range), N (%)**
**Bone mineral density (hip)**	0.82 (0.76 - 0.92) g\cm^2^
**Vertebral T score**	-1.41 ± 0.13
**Hip T score**	-0.91 ± 0.095
**Vertebral osteoporosis**	
**Classification**	50 (50%)
Normal	29 (29%)
Vertebral osteopenia	21 (21%)
Vertebral osteoporosis	
**Hip osteoporosis classification**	
Normal	72 (72%)
Hip osteopenia	23 (23%)
Hip osteoporosis	5 (5%)

*Abbreviations*: *SD* standard difference.

### Fracture risk assessment tool

Using FRAX calculator with BMD data, the median 10-year (interquartile range) probability for MOF was 3.7% (2.43 – 6.18) and that for HF was 0.3% (0.10 - 0.68). Two participants were at high risk of MOF (≥20%) and 4 were at high risk of HF (≥3%). Based on the NOF guideline, at least 24 participants needed immediate treatment. Table 3 shows data extracted from FRAX calculator. BMD was significantly and negatively correlated with both 10-year probability of MOF (p <0.001, r = - 0.609) and HF (p <0.001, r = - 0.845).

**Table 3:** **Association and correlation between 10**-**year probability of MOF and HF with demographic and clinical variables of the participants**

**Variable name**	**Median (interquartile range) 10-y prob. of MOF; ( *P value *)**	**Median (interquartile range) 10-y prob. of HF; ( *P value *)**
**Age category**		
50 < Age ≤65	3.2% (2.20 – 6.08)	0.2% (0.00 – 0.48)
90 ≥ Age >65	4.9% (3.65 – 6.50)	0.5% (0.4 – 0.98)
	P = 0.008^a^	P < 0.001^a^
**Gender**		
Male	3% (1.65 – 4.65)	0.2% (0.00 -0.45)
Female	3.8% (2.70 – 6.5)	0.4% (0.10 - 0.7)
	P = 0.024^a^	P = 0.087 ^a^
**Marital status**		
Married	3.5% (2.3 – 6.2)	0.3% (0.1 – 0.6)
Others	4.5% (3.2 – 5.55)	0.6% (0.25 – 0.95)
	P = 0.47^a^	P = 0.088^a^
**Parity**		
Null parity	3.8% (2.58 - 4.4)	0.5% (0.15 – 0.6)
Have ≤ 6 children	3% (2.30 – 5.3)	0.1% (0.08 – 0.4)
Have > 6 children	5.3% (2.95 – 7.5)	0.5% (0.25 – 1.0)
	P = 0.003^b^	P = 0.002^b^
**Education level**		
Illiterate	5.4% (3.78 – 7.35)	0.55% (0.4 – 1.05)
School educated	3.4% (2.3 – 6.28)	0.3% (0.1 – 0.7)
Achieved university degree	2.9% (2.15 – 3.98)	0.2% (0.1 – 0.4)
	P = 0.01^b^	P = 0.005^b^
**Current tobacco use**		
Yes	3.4% (2.6 -5.6)	0.4% (0.2 – 0.7)
No	3.8% (2.3- 6.4)	0.3% (0.1 – 0.6)
	P = 0.802^a^	P = 0.199^a^
**BMI**	-	-
	P = 0.576^c^, r = 0.057	P = 0.798^c^, r = -0.026
**Exercise**		
Yes	3.1% (1.88 – 5.7)	0.2% (0.00 – 0.63)
No	3.8% (2.78 – 6.35)	0.4% (0.1 – 0.73)
	P = 0.066^a^	P = 0.098^a^
**Previous fracture**		
Yes	6.75% (5.58 - 10.45)	0.7% (0.38 – 1.43)
No	3.2% (2.2 - 4.8)	0.2% (0.1 – 0.5)
	P < 0.001^a^	P < 0.001^a^
**Previous parents’ hip fracture**		
Yes	7.2% (5.7 -13)	0.3% (0.3 – 1.1)
No	3.3% (2.3 – 5.3)	0.3% (0.1 – 0.65)
	P < 0.001^a^	P = 0.211^a^
**History of OCT use**		
Yes	3.4% (2.45 – 5.33)	0.3% (0.10 – 0.63)
No	3.9% (2.9 – 7.2)	0.4% (0.15 – 0.8)
	P = 0.031^a^	P = 0.115^a^
**History of corticosteroid use**		
Yes	6.7% (5.2 – 10.7)	0.55% (0.2 – 1.03)
No	3.05% (2.2 – 4.83)	0.3% (0.1 – 0.5)
	P < 0.001^a^	P = 0.031^a^
**Secondary osteoporosis**		
Yes	5.4% (3.9 – 7.2)	0.5% (0.3 – 1.2)
No	3.4% (2.3 – 5.65)	0.3% (0.1 – 0.6)
	P = 0.013^a^	P = 0.034^a^
**Diabetes mellitus**		
Yes	4.9% (2.75 – 6.5)	0.4 (0.2 – 0.95)
No	3.4% (2.30 – 5.5)	0.3 (0.1 – 0.5)
	P = 0.12^a^	P = 0.024^a^
**Cardiovascular diseases**		
Yes	3.9% (2.5 – 6.18)	0.4% (0.1 – 0.7)
No	3.3% (2.3 – 6.05)	0.3% (0.1 – 0.6)
	P = 0.418^a^	P = 0.665^a^
**Gastrointestinal tract**		
**Disorders**	4.9% (2.9 – 6.68)	0.5% (0.13 – 0.95)
Yes	3.35 (2.3 – 5.93)	0.25% (0.1 – 0.5)
No	P = 0.09^a^	P = 0.031^a^
**Rheumatoid arthritis**		
Yes	5.3% (3.65 – 7.5)	0.6% (0.4 – 1.1)
No	3.3% (2.30 – 5.6)	0.2% (0.1 – 0.5)
	P = 0.002^a^	P = 0.003^a^

*Abbreviations*: *SD* standard difference, *Q*1-*Q*3 quartile 1, quartile 3, *OCT* oral contraceptives, *MOF* major osteoporotic fracture, *HF* hip fracture, *BMI* body mass index.

^a^Statistical significance of differences estimated with the Mann–Whitney *U* test.

^b^Statistical significance of differences estimated with the Kruskal-Wallis test.

^c^Statistical significance of differences estimated with the Spearman's correlation coefficient.

The median 10-year probability of MOF calculated based on BMD was significantly (p <0.05) associated with age > 65 years, female gender, patients who have more than 6 children, low educational level, history of previous fracture, history of parents’ HF, history of no OCT use among females, history of oral corticosteroid use, secondary osteoporosis, and presence of rheumatoid arthritis but not with marital status, smoking, BMI, performing exercise, diabetes mellitus, and presence of cardiovasular diseases (CVD) or gastrointestinal tract (GIT) diseases.

The median 10–year probability of HF calculated based on BMD was significantly (p <0.05) associated with age > 65 years, patients who have high number of children (more than 6 children), low educational level, previous HF, secondary osteoporosis, use of corticosteroids, secondary osteoporosis, presence of GIT diseases and rheumatoid arthritis but not with gender, marital status, current tobacco smoking, BMI, exercising, previous history of parents’ hip fractures, history of OTC use, and history of CVD.

## Discussion

The problem of osteoporosis will soon be of greater importance in developing countries due to the increase in life expectancy [11]. In the Middle East, the burden of this disease is expected to increase taking into account the steady growth of the ageing population. A study in Iran indicated that 2 million people are at risk of fracture and the cost of HF is between 6 – 8 million United Sates dollars (USD) [12]. The prevalence of osteoporosis in post-menopausal Iranian women was reported to be 6 percent which is remarkably low compared to other countries. A recent survey conducted in Lebanon to determine risk factors for osteoporosis in the Lebanese female population found that back pain, low physical activity, family history of osteoporosis or HF, loss of height, early menopause, heavy smoking (>20 cigarettes per day), thin and small build, history of rheumatoid or thyroid disease, previous administration of corticosteroids and chronic alcohol consumption were associated with increased MOF [13]. In Saudi Arabia, the prevalence of osteoporosis was studied in a group of randomly selected males and females aged 20–79 years; the prevalence in women was 28.2% [14]. In a study carried out in Qatar on healthy females aged 20 to 70, risk factors for osteoporosis were similar to those known to influence BMD in other populations; female sex, age, early menopause, and smoking [15]. In Palestine, a study conducted by Abd-Alhameed et al. (2010) on the prevalence and awareness to osteoporosis among randomly selected post-menopausal women found that osteoporosis at lumbar spine, neck and total hip was 24%, 14% and 29.7% respectively [16]. The authors of the Palestinian study concluded that BMD values declined 0.32-0.53% per year in relation to the number of years after menopause.

Several studies in Europe and Asia were carried out to assess the 10-year risk probability for bone fracture using FRAX but none were carried out in Arab world. A study in Bulgaria among women > 50 years found that the mean 10-year absolute fracture risk was 13.4 ± 9.2% (major fractures) and 2.8 ± 5.2% (HP) [17]. A study in Taiwan found that the mean 10-year probabilities of MOF or HF were 13.8% (95% confidence interval (CI) = 10.7%-16.9%) and 2.2% (95% CI = 0.8%-3.5%), respectively [18]. A study in Poland among 2012 post-menopausal women found that the mean 10-year probability of MOF or HF was 22.2 ± 12.1% [19]. It is evident that the values obtained in our study were lower than that reported in other international studies. This difference could be attributed to geographical, cultural and nutritional habits in different parts of the world.

Osteoporosis is a major public health problem because of the fractures that could occur. Unfortunately, osteoporosis receives low attention in the primary health care programs in most underdeveloped countries where most women are largely unaware of the serious complications associated with osteoporosis [20]. Evidently, minimizing the risk of acquiring the disease begins by modification of individuals’ life style to combat related risk factors and identification of patients at high risk to reduce future fractures. Many risk factors, some are modifiable and others non-modifiable are associated with osteoporosis. The major non-modifiable risk factors include advanced age, a personal history of fractures as an adult, and a history of fracture in a first degree relative [21,22]. Major modifiable risk factors include a low BMD, chronic oral corticosteroid use (more than 3 months of use), history of recurrent falls, and a low body weight (less than 58 kg) [21-24]. Minor risk factors for MOF include, but are not limited to, inadequate nutritional supplementation of vitamin D and calcium, impaired eyesight despite correction, high alcohol and tobacco consumption, and immobilization [7,25,26].

Results of our study showed a good agreement with published studies regarding risk factors that are significantly associated with risk of fractures. However our results showed no significant association between the risk of fractures and BMI or regular exercise. In our study, the 10-year probability of MOF was significantly associated with higher age, female gender and multi parity. Our results are consistent with results published in literature where similar significant association between risk of osteoporosis and such variables was found [15,21]. Previous fracture, presence of secondary osteoporosis, rheumatoid arthritis, and administration of corticosteroid were also significantly associated with 10-year probability of HF and MOF. Similar results were found in literature [21,23,24]. Smoking, exercising, and having a history of cardiovascular diseases were not significantly associated with 10-year probability of MOF. This may be due to the small sample size which made it difficult to obtain statistical difference. Gastrointestinal diseases and diabetes mellitus were significantly associated with 10-year probability of HF. This may be due to their contribution to occurrence of secondary osteoporosis in this region, increasing the rates of HF.

Our study has few limitations. First the sample was limited to one district. It would be better if we could have sample from the whole west bank, but high cost of the DEXA was one of the barriers. Furthermore, the sampling was carried out at specific times, dates and on people attending a healthcare clinic. This might suggest that most of those participants were not healthy adults and this might affect the interpretation of our results and might limit the generalization of our results to the entire Palestinian population. In Palestine, recruitment of participants for such study is a very difficult task given the cultural barriers as well the nature of the test which requires certain procedures that might not be acceptable for some people. Second, the majority of the sample was females and obese people. Obesity is common among Palestinians particularly women [27]. This might be due to decreased physical activity and greater than necessary food consumption. In addition, leisure-time physical activity is not a common concept in the Palestinian context, especially for rural women, where lack of sex-segregated facilities and cultural norms are prohibitive factors. Women in urban areas face similar cultural restrictions [27]. The prevalence of obesity among participants might have increased the 10-year probability estimation of bone fractures. Third, participants might have made errors related to recalling certain events or understanding certain questions. For example, most participants were not able to report accurately the presence of rheumatoid arthritis. Participants reported a history of joint pain which might be rheumatoid arthritis or osteoarithritis. However, we analyzed information related to history or current joint pain as if it is rheumatoid arthritis.

## Conclusions

As a conclusion, our study indicated that osteoporosis is common among the study sample where one fifth of the participants had vertebral osteoporosis and 5% had hip osteoporosis. The median 10-year probability of MOF was higher than that of HF and both were within reported range of published results. There is an urgent need of a comprehensive national program to screen for osteoporosis in Palestine. More care and attention should be targeted toward elderly and especially postmenopausal female with respect to preventive measures. More efforts on the level of MOH to adopt FRAX tool to be used as a screening tool for all individuals above 40 years before making DEXA as an approximate estimation of fracture risk is a priority. Increase awareness toward osteoporosis and prevention strategies among the general population and particularly in older population. Further research and studies regarding fracture rates, genetic component of osteoporosis, and evaluation of the applicability accuracy and feasibility of FRAX in Palestinian population are needed.

## Abbreviations

FRAXm: Fracture risk assessment (FRAX) tool; WHO: World health organization; MOF: Major osteoporotic fracture; MOH: Ministry of health; HF: Hip fracture; DEXA: Dual energy X-ray absorpitometry; BMD: Bone mineral density; IRB: Institutional review board; SPSS: Statistical package for social sciences; UK: United Kingdom; Q1-Q3: Lower – upper quartiles; SD: Standard deviation; OR: Odds ratio; CI: Confidence interval; RA: Rheumatoid arthritis; USA: United States of America; CVD: Cerebrovascular disease; BMI: Body mass index; USD: The United States dollar; GIT: Gastrointestinal tract.

## Competing interests

The authors declare that they have no competing interests.

## Authors’ contributions

All authors were involved in drafting the article and all authors approved the final version to be submitted for publication. All authors have added an intellectual significant value to the manuscript. MA was involved in subject recruitment and interview, data collection, data coding and entry, literature review, data analysis, data interpretation and manuscript editing. This was done in partial fulfillment of a master degree in Public Health program at An-Najah National University. AA was involved in concept, study design, manuscript writing and editing, and academic co-supervision for MA according to An-Najah university regulations. SZ and AS were involved in concept, study design, intellectual critique and data interpretation. SA was involved in concept, study design, critical editing and revision of the manuscript. WS was involved in the study conception and design, literature review, manuscript writing, manuscript submission, manuscript revision, head of the research group, and academic supervision for MA according to An-Najah university regulations. This manuscript is part of a project for master degree in the graduate program of public health. The project was initially and originally conceptualized and designed by the Clinical Pharmacology/ Toxicology Research Group at An-Najah National University (WS, AA, SZ, AS and SA). The project was then assigned to MA as a thesis project and was academically supervised by WS and AA in adherence to An-Najah University regulations with regard to academic supervision for graduate students. The project was successfully defended as a master thesis by MA at An-Najah University on Feb 2013. A copy of the full thesis is available at An-Najah National University library.

## Authors’ information

Professor Waleed M. Sweileh is the head of a research group (S.Z, S.A, A.S and A.A) which has published in the field of clinical pharmacology, toxicology, pharmacoepidemiology, social and community pharmacy, clinical pharmacy and medicine. The research group has also supervised many students in the fields of nursing, public health and pharmacy.

## Pre-publication history

The pre-publication history for this paper can be accessed here:

http://www.biomedcentral.com/1471-2474/14/284/prepub
